# Estimation of Respiratory Volumes During Tidal Breathing Using Two Depth Cameras

**DOI:** 10.3390/s26185793

**Published:** 2026-09-12

**Authors:** Adam Handley, Stephen Preece, Phil Tresadern

**Affiliations:** 1 School of Health and Society, University of Salford, Salford M6 6PU, UK; 2 Visionomy, Salford M30 9HS, UK

**Keywords:** optoelectronic plethysmography, depth camera, RGB-D cameras, non-contact respiratory monitoring, respiratory diagnostics, spirometry

## Abstract

**Highlights:**

**What are the main findings?**

- A system of two facing depth cameras can accurately estimate breathing volumes.

**What are the implications of the main findings?**

- Depth cameras could be used instead of a laboratory motion capture system for optoelectronic plethysmography applications.
- Depth cameras offer the potential for clinical implementation of respiratory monitoring.

**Abstract:**

Recent advances in depth camera technology enable precise quantification of shape changes of the human torso during breathing and therefore predict respiratory volumes. The aim of this study was to investigate the potential of estimating tidal volumes during relaxed breathing from two depth cameras. Data were analysed from 21 healthy participants. For each participant, a set of 30 physical markers were placed across the torso and tracked using two depth cameras. Marker positions were mapped to 3D coordinates and used as inputs for a volume algorithm that estimated respiratory volumes. A breath-by-breath analysis was then conducted to compare estimated volumes with spirometry measurements. The camera-derived volumes were strongly correlated with spirometry (marginal R^2^ = 0.941) and showed excellent agreement with a mean (SD) absolute difference of 78.0 (34.8) mL. Bland–Altman analysis showed a bias of −4.6 mL and 95% limits of agreement of −192.3 to 183 mL. The reduced 17-marker configuration also showed a strong correlation with spirometry (marginal R^2^ = 0.918) but reduced agreement, with a mean (SD) absolute difference of 107.4 (58.2) mL and a Bland–Altman bias of −76.4 mL (95% limits of agreement: −298.1 to 145.3 mL). This is the first study to use a marker-based system to estimate respiratory volumes from two facing depth cameras. The use of markers allows for segmentation of the torso and therefore offers the potential to characterise breathing without the need for an expensive laboratory motion capture system.

## 1. Introduction

Measuring respiratory function is of critical importance in the diagnosis and monitoring of respiratory disease. The gold-standard measure is spirometry, in which patients are required to perform specific breathing tests while the device measures volume and flow of the inspired and expired air [1]. Based on these variables, clinicians can draw conclusions about restriction and obstruction of the airways. Measurements of respiratory function are also incorporated within cardiopulmonary exercise testing (CPET), a maximal exercise assessment that evaluates cardiovascular and pulmonary responses to exertion. CPET has a wide variety of uses in medicine, such as determining surgery risk, cardiorespiratory pathophysiology and exercise prescription as well as applications to assess fitness for competitive athletes [2]. However, a critical limitation of respiratory monitoring is the need for the patient to wear a facemask during the test. This can be uncomfortable, can increase airflow resistance and may increase infection risk.

Camera-based systems offer an alternative method for quantifying respiratory function by using volume measurements of the upper body to estimate flow and volume during breathing. This technique is known as optoelectronic plethysmography (OEP) [3] and, until recently, was only possible using motion capture camera systems that use infrared light to track reflective markers in 3D space. With this approach, a system of 89 reflective markers is placed in a grid-like pattern across a participant’s chest and back and tracked with a system of 8 to 12 cameras that are positioned at different viewing angles [4,5]. In addition to estimating flow and volume, OEP can be used to characterise breathing pattern, for example, abdominal–thoracic synchrony. This synchrony can be used to provide real-time feedback on respiratory control, which can then facilitate breathing retraining [6]. However, despite its widescale use in research, OEP has not been adopted into clinical practice because of high costs, challenges associated with camera set up and the time-consuming process of placing and tracking 89 markers across the body.

Recent advances in sensing technology now mean that it is possible to capture 3D information during human breathing using inexpensive depth cameras [7,8]. Several studies have sought to understand the potential of using this technology to characterise respiratory function. This work includes studies aimed at estimating both breathing frequency [9,10,11,12,13,14] and breathing volumes [15,16,17,18,19,20,21,22,23,24]. However, while estimation of frequency is relatively straightforward, estimation of accurate tidal volumes [15,16,17,18,19,20] and/or lung function parameters [21,22,23,24] required the use of complex calibration or scaling techniques [15,19,21,22,24]. This is because it is difficult to differentiate between changes in torso shape, which occur with breathing, and postural sway [15,23] when using a single camera. This challenge means that previous research with a single camera has reported only moderate agreement with gold-standard spirometry.

To date, only one previous study has investigated the possibility of using a system of two facing depth cameras to estimate respiratory volumes [23]. This approach is an advancement on the use of a single depth camera because motions of the anterior and posterior aspects of the torso can be quantified simultaneously, enabling accurate characterisation of breathing motions. Indeed, the study by Soleimani et al. [23] demonstrated that a dual-camera approach could deliver substantially higher accuracy than the single-camera approach. However, their method required the use of an individual calibration using spirometer-measured volumes, limiting potential clinical applicability. Furthermore, because the analysis was performed using point cloud data rather than a grid of markers defined from anatomical landmarks, this approach may not allow for characterisation of breathing pattern using established OEP techniques [3].

Given the limitations of previous research, we sought to investigate the possibility of using two depth cameras to implement OEP respiratory measurement and characterisation [4]. Specifically, we wanted to understand if it would be possible to use two facing RGB-depth sensors to obtain data on a set of 89 markers. To minimise set up time, we investigated the possibility of reducing the number of physical markers by interpolating across the point cloud and using regression to define a set of virtual markers.

## 2. Methods

### 2.1. Participants

Twenty-four participants took part in the study (10 female), mean (SD) height: 1.74 (0.1) m, mean (SD) weight: 73.5 (15.1) kg and mean (SD) BMI: 24.3 (4.1) kg/m^2^. Ethical approval was provided by the NHS Health Research Authority (21/EM/0221), and all participants provided informed consent. Participants were eligible if they were aged 18 years or older and reported either no respiratory condition or mild obstructive asthma that did not impair normal, relaxed breathing. Individuals were excluded if they had received treatment for an acute respiratory tract infection within the last 3 months. During data collection, participants were instructed to stand in a relaxed posture and breathe normally through a spirometer while two 3D cameras recorded movements of the torso.

### 2.2. Set up

Two Intel RealSense L515 cameras (RealSense, Cupertino, CA, USA) were positioned 2 m apart such that the participant could stand between them, approximately 0.8 to 1.0 m from each camera origin. The cameras’ heights were adjusted so that they were at the height of the centre of the chest to ensure whole-torso visibility, and the cameras were positioned in portrait orientation to maximise visibility of the torso. The two L515 cameras (see [25] for technical specification) were connected to a laptop (Republic of Gamers Zephyrus G15, AMD Ryzen 9-6900HS, 32 GB RAM, Republic of Gamers, Taipei, Taiwan), each via an internal USB root hub using Newnex 5 m 5 Gb/s USB 3.1 Gen 1 A to C cables (Newnex, Santa Clara CA, USA). Recordings were taken using the Intel RealSense Viewer (v2.50.00) at 30 Hz with the default short-range settings. Each camera recording consisted of an RGB stream and a synchronised depth stream.

Software provided by the camera manufacturer facilitated mapping between pixels in the RGB image and the corresponding 3D coordinates in the depth stream using the camera’s intrinsic calibration parameters [26], a process we refer to as pixel-point mapping.

### 2.3. Defining a Global Coordinate System

**Figure 1:**
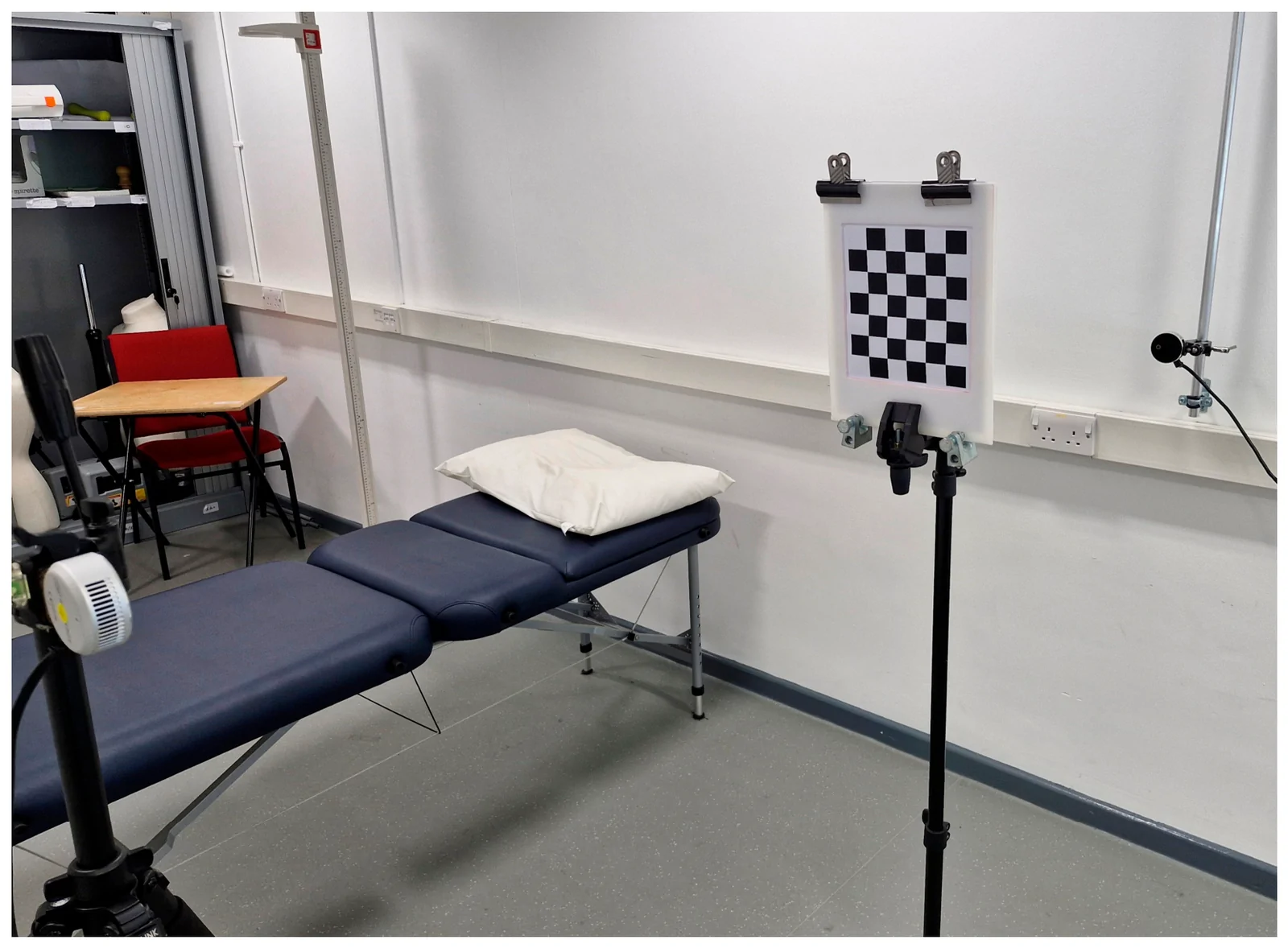
Pose estimation using a double-sided checkerboard calibration grid placed between the two L515 cameras.

A two-stage process was used to define a global coordinate system before collection of respiratory data. For the first stage, a 6 × 7 double-sided checkerboard, acting as a calibration grid [23], was positioned equidistant between the two cameras (Figure 1). The grid was identical on each side and was of negligible thickness; therefore, grid intersection points were equivalent on each side. These intersection points acted as calibration points that were used to transform from the coordinate frame of each individual camera to a global frame. Using data from a 10 s recording, the grid intersection points were identified from the RGB camera stream using OpenCV (4.7.0.68). These pixels were then mapped to corresponding 3D coordinates in the depth stream using pixel-point mapping, as described above. To minimise the effect of depth measurement variations, the mean 3D position for each intersection point was calculated over the 10 s trial. A transformation matrix was then obtained for each camera using a least-squares regression fit between the coordinates of the intersection points in the camera’s frame and corresponding coordinates in the checkerboard frame of reference. These transformation matrices defined the local to global transformation for each camera.

**Figure 2:**
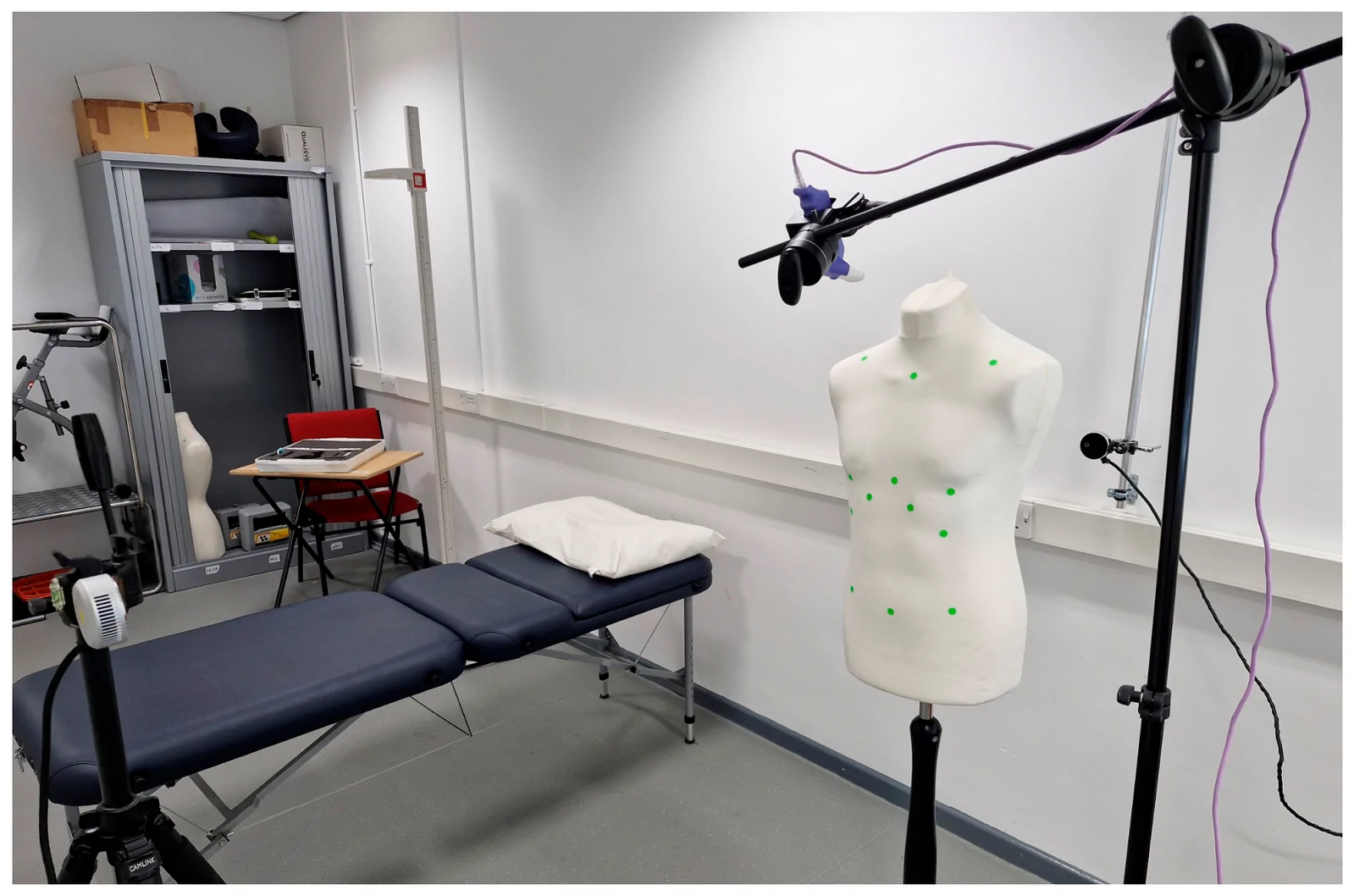
Mannequin used to refine the transformation matrices and custom rig for mounting the spirometer at mouth height.

In the second stage, the two transformation matrices were refined using a set of 17 known reference points on a mannequin (Figure 2). The reference points were first defined using a Shining 3D scanner (Handheld Rapid Scan with high resolution; accuracy: 0.5 mm, point distance: 0.5–3.0 mm. EinScan-Pro, Shining 3D Tech Co., Ltd., Hangzhou, China) that enabled the precise relative position of each marker to be determined. The Shining 3D scanner was calibrated for a scanning distance of 350–450 mm, and scanning distance remained within this range. For the calibration, a 10 s recording of the mannequin was obtained using the L515 and the same process described above used to calculate 3D coordinates of each marker in each camera’s frame of reference. These coordinates were then transformed to the global frame using the transformation matrices and compared to the reference coordinates. A least-squares regression fit was then used to refine the scaling component of the transformation matrices to optimise the match with calibration points.

### 2.4. Experimental Data Collection

**Figure 3:**
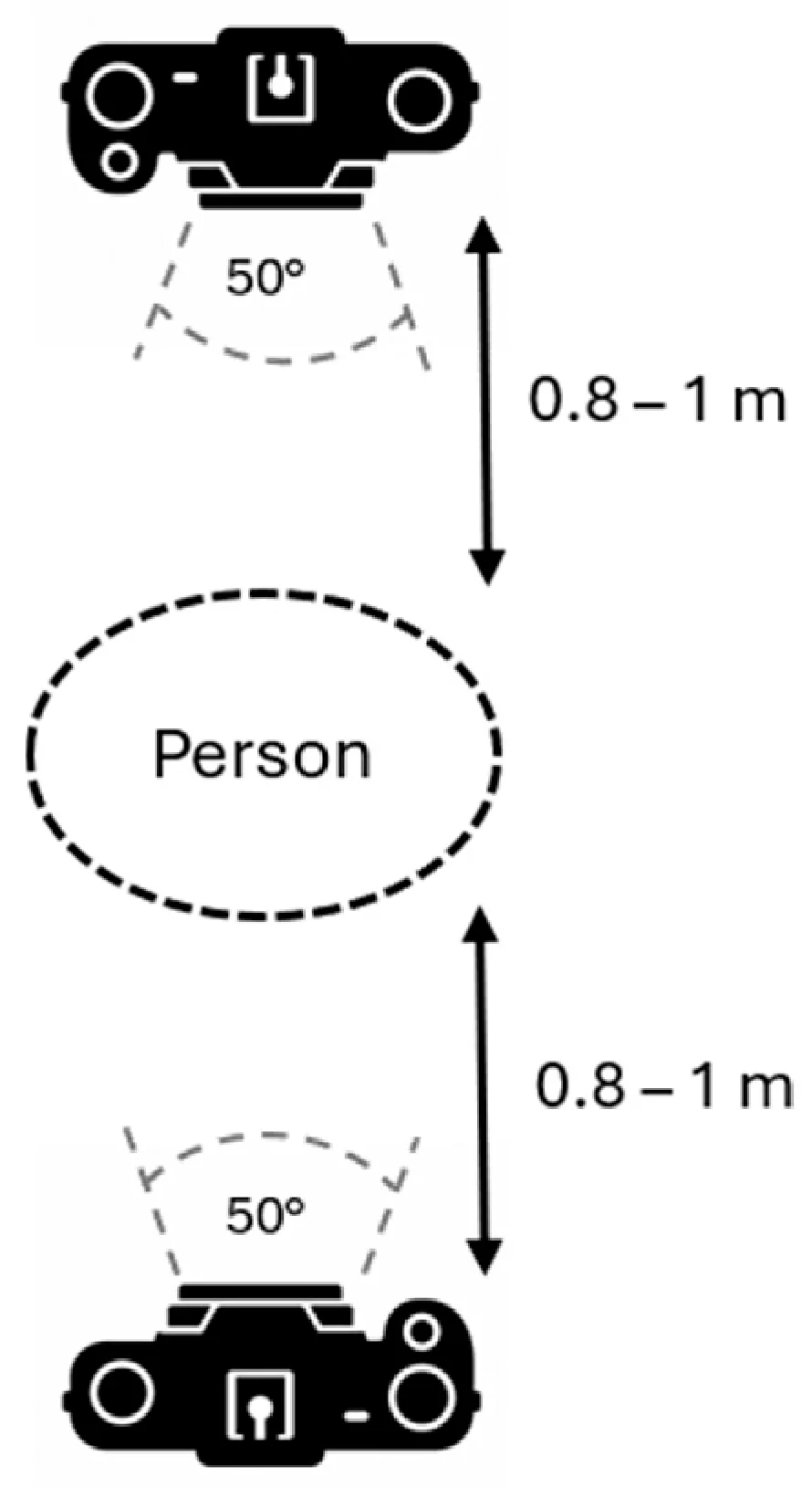
Schematic to illustrate camera positioning during data collection.

Participants stood unclothed from the waist upwards (male) or wearing a sports bra (female) between two cameras (Figure 3). Spirometry was recorded using the Easy-on PC Spirometer (ndd Medical, Zurich, Switzerland) and calibrated according to the manufacturer’s instructions. The spirometer was mounted in a custom rig at a comfortable position for the participant’s mouth (Figure 2) and connected to a second laptop (MSI Stealth GS66, Intel i9-12900H, 32 GB RAM, MSI, New Taipei City, Taiwan). Flow and volume data were recorded in WBreath (v4.0.15.0, ndd Medical, Zurich, Switzerland) at 50 Hz.

**Figure 4:**
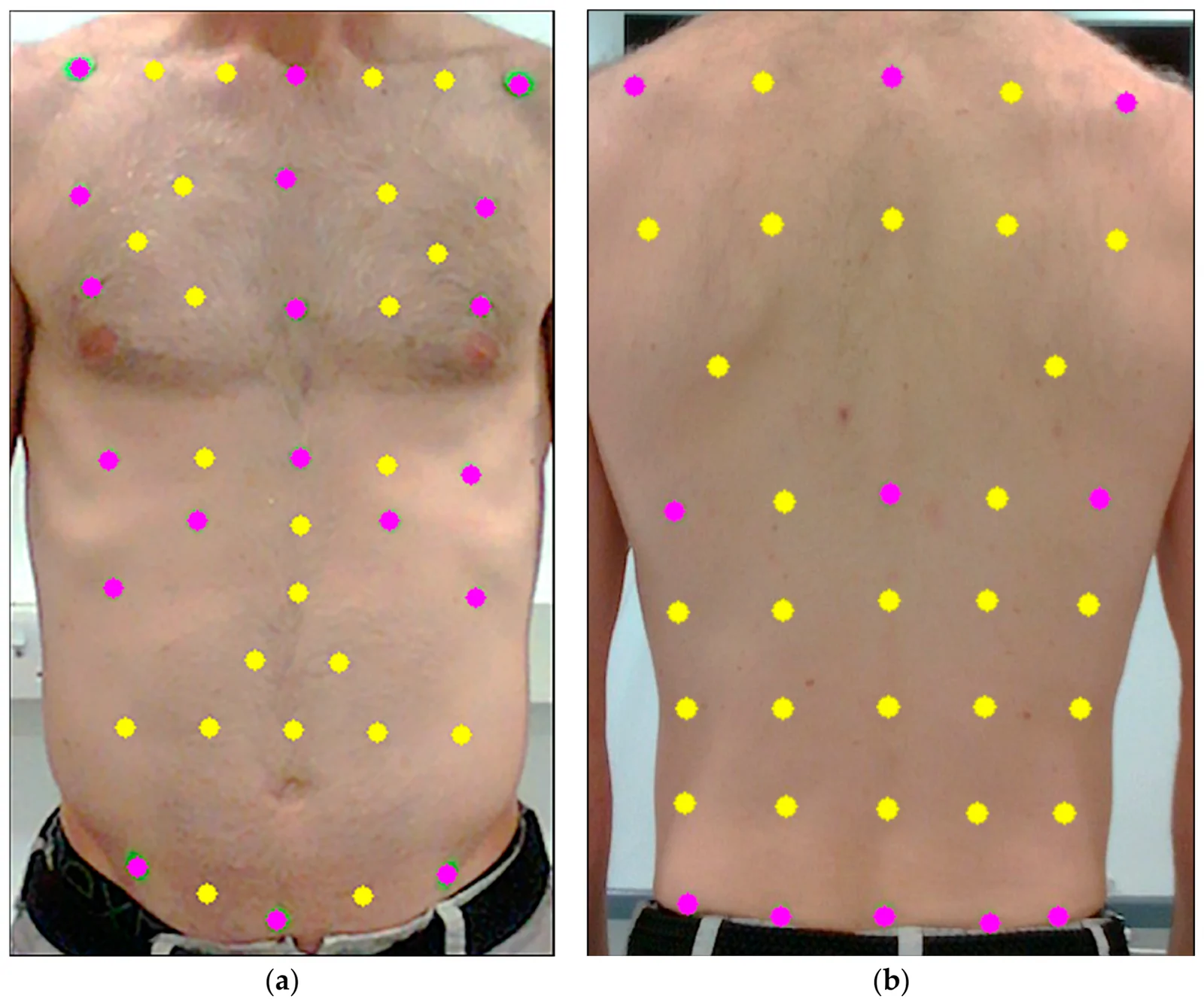
Physical (pink) and virtual (yellow) markers identified using the tracking software on the anterior (**a**) and posterior (**b**) torso.

Traditional OEP approaches for estimating respiratory volumes use a set of 89 reflective markers placed across the torso [4]. As explained earlier, this allows for anatomical segmentation and characterisation of breathing pattern. Our aim was to generate an equivalent dataset by combining a small set of markers placed on the torso with a set of virtual markers obtained via interpolation across the point cloud (described later). A set of 30 stickers (13–19 mm diameter) were placed on the torso in the configuration shown in Figure 4, with 19 on the chest and 11 on the back. These markers were chosen as a minimum subset of the 89 used for OEP [3], which allowed for anatomical definition of the pulmonary ribcage, the abdominal ribcage and abdomen. Placing the markers took approximately 5 min per participant.

After marker placement, camera data and spirometry data were recorded during normal tidal breathing. Participants wore a nose clip to ensure all air flow passed through the spirometer mouthpiece. Each trial lasted 30 s and consisted of normal, tidal breathing followed by a large synchronisation breath. Each participant completed 3–5 trials to ensure at least 10 tidal breaths were captured for analysis.

### 2.5. Volume Algorithm

**Figure 5:**
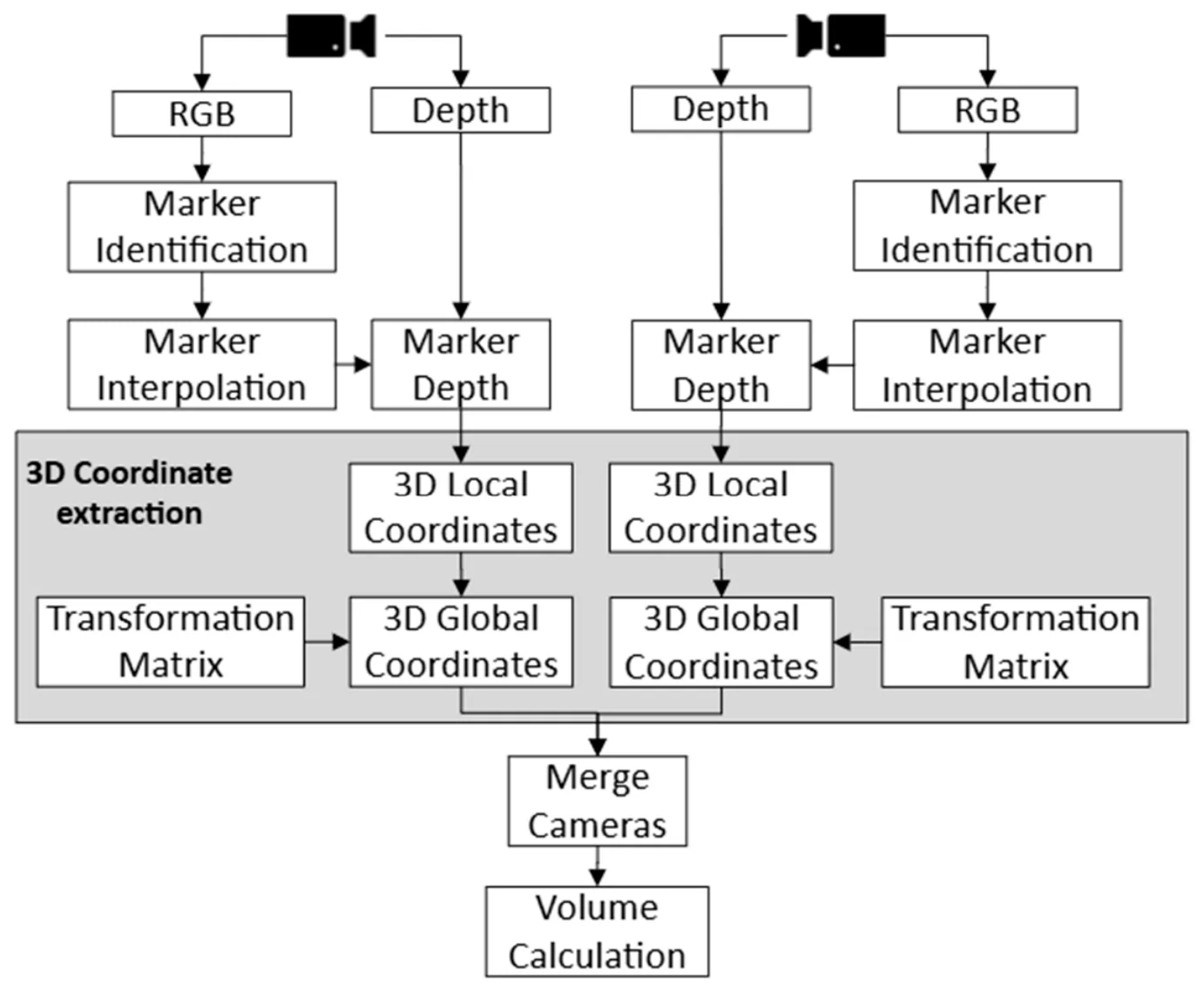
Processing pipeline for the volume algorithm.

Figure 5 provides an overview of the volume algorithm. For each camera, the physical markers were identified in the RGB stream by applying HSV ranges. Specifically, each frame was converted from RGB to HSV, and masking applied to pixels outside of the HSV range. OpenCV blob detection, incorporating morphology and connected component analysis methods, was then used to compute the centroid of each marker in the masked binary image. The parameters for the blob detection were individualised for each trial to ensure that all markers were correctly identified. The parameters that were adjusted included minimum area, minimum distance between the markers, HSV ranges and region of the image to search. Additionally, a depth filter was applied to ensure only pixels were identified within 1.5 m from the camera origin. Once the correct number of markers were identified in each frame, labelling was performed by systematically ordering the centroids in the horizontal and vertical direction.

Once the 30 physical markers were labelled, virtual markers (shown in yellow in Figure 4) were estimated using interpolation. This was achieved by defining the pixel location of each virtual marker as a geometrical relationship from a set of surrounding physical markers (see Appendix A for details). Using this process, we defined a set of 49 virtual markers. The final set of 79 markers formed a grid of points across the torso, which were mapped to a corresponding 3D coordinate in the camera’s frame of reference using pixel point mapping. Coordinates were then transformed into the global system using the transformation described earlier (Section 2.3). The process was repeated at each time point, and data from the front and rear cameras were aligned in time using the sample timestamp.

To recreate the full set of 89 markers used in the traditional OEP approach, it was necessary to estimate the position of a further 10 markers. These markers are positioned on the lateral aspects of the torso at the midaxillary line [27] and therefore could not be captured using a system of two facing depth cameras. To estimate the 3D coordinates of these 10 markers, we developed a regression model that was based on data from the full set of 89 markers positioned on the mannequin shown in Figure 2. Specifically, we used least squares regression to define a mapping from the set of 79 measured markers to the equivalent set of 79 markers on the mannequin. This mapping involved optimising translation, rotation and scaling in each plane. Once defined, this mapping allowed us to estimate the positions of the final set of 10 markers from the mannequin data. The least squares fitting process was performed for every time frame in each trial. Without this regression approach, it is not possible to capture the full 3D geometry of the torso, and this leads to a systematic underestimate of torso volume (see Appendix B).

The final trajectory of each of the 89 markers was filtered using a 4th-order low-pass Butterworth filter at 2 Hz. Tidal breathing volumes were then calculated using the 89 marker trajectories using a published algorithm [4]. Specifically, markers were divided into layers, and a centroid was calculated for each layer. Each layer was then divided into a series of tetrahedrons. The volumes of 292 tetrahedra were summed to give the total volume of the chest. With this approach, volume calculations are invariant to small between-participant differences in precise marker locations. Unfortunately, the LibRealsense has persistent issues with frame drop (Issues #588, #9385, #11530, #11383 [26]). Therefore, any trials in which there was frame drop longer than 5 s or in which there was frame drop during the synchronisation breath were excluded. For recordings containing frame drops longer than 1 s or frame drop occurring at a turning point, the affected breath was excluded from the analysis. Frame drops were identified from gaps in the recorded frame timestamps. All exclusions were performed prior to comparison with spirometry data.

Alongside the volume calculation using the set of 30 physical markers, we investigated whether it would be possible to estimate volumes using a reduced set of markers. A subset of 17 of the original 30 physical markers were selected on the basis that they could be used to segment the torso. Following a similar interpolation approach, we defined a set of 62 virtual markers (Appendix A) using the same datasets used for analysis of the full marker set. Once the 79 markers had been defined, we followed the same process to obtain 3D coordinates in the global frame, regression to define the remaining 10 marker coordinates and Butterworth filtering smooth individual marker trajectories.

All processing and volume calculations described above were written in python 3.10. The participant data and necessary python code for calculating and analysing the volumes presented in the study are openly available in Figshare at https://doi.org/10.17866/rd.salford.32587968.

### 2.6. Spirometry Comparison and Statistical Analysis

Spirometer volumes were exported directly from Wbreath and aligned with the camera data via the following steps. Firstly, volume data were resampled to 50 Hz to match the spirometry data using spline interpolation. Secondly, cross-correlation analysis between the camera and spirometer volumes was used to determine the time lag at the peak correlation. The synchronisation breath ensured that the highest correlation was found when the largest volume changes were observed in both signals. This lag was then used to synchronise the signals, after which the synchronisation breath was removed.

Analysis was performed on a breath-by-breath basis using a peak detection algorithm. For each breath, expired tidal volumes (TVs) were determined as the difference between the end-inspiratory volume and the end-expiratory volume. Peaks were identified in both spirometer and camera volumes using the Python SciPy (v1.11.4) function find_peaks with peak prominence of 0.05 and width of 10. As the camera and spirometer signals were temporally synchronised by cross-correlation lag, turning points were identified independently in each signal using find_peaks, and corresponding breaths were then matched using a nearest-neighbour approach based on the temporal proximity of turning points in the two signals. Absolute differences and root-mean-squared error (RMSEs) between the TVs were assessed. To facilitate comparison with Massaroni et al. [4], the normalised Euclidean d and percentage discrepancies were also calculated using Equations (1) and (2) provided below.

(1) $$d=\frac{{\left|TV_{Spirometry}-TV_{Camera\;method}\right|}^{2}}{\left|TV_{Spirometry}\right|\left|TV_{Camera\;method}\right|}$$

(2) $$\%discrepancy=\frac{TV_{Camera\;method}-TV_{Spirometry}}{TV_{Spirometry}}\cdot 100$$

We conducted linear mixed-effects model correlation analysis with an additional first-order autoregressive (AR(1)) residual correlation structure and a repeated measures Bland–Altman analysis. Because breaths within a trial are temporally ordered and physiologically coupled, we validated whether a combined random-intercept and AR(1) model was required (see Appendix C). The final linear relationship between OEP and spirometer tidal volume was estimated using a mixed-effects model (TV_camera_ ~ TV_spirometer_, random intercept + AR(1) for participant, restricted maximum likelihood) fitted in R (v4.6.1). Marginal R^2^ (fixed effects only) and conditional R^2^ (fixed and random effects combined) were calculated following Nakagawa and Schielzeth [28]. The bias, between-subject and within-subject variance components were taken directly from the model, and limits of agreement were calculated as described by Bland and Altman [29].

The analysis was repeated separately for the full marker set and the reduced marker set. Analyses were performed in Python 3.10 and R 4.6.1 (nlme). Claude Code (v2.1.153) was used for generating python code for the comparison, statistical analysis and producing figures with Matplotlib (v3.7.1). All code produced by Claude Code was manually checked and verified using data with known properties.

## 3. Results

Three participants (two female) were excluded from the analysis due to persistent frame drop. The frame drops were observed in both the front and back cameras, indicating that they were not attributable to a fault in a single device. The remaining twenty-one participants were included in the final analysis (eight females). There was a close match between the camera-derived tidal volumes and spirometry for the full marker set. For this analysis, a total of 515 breaths were identified across all 21 participants, with a mean (SD) in the absolute difference of 78 (34.8) mL and mean (SD) RMSE of 90.2 (34.4) mL (Table 1). Differences between the camera and spirometer were larger for the reduced marker set, with a mean (SD) in the absolute difference of 107.4 (58.2) mL and mean (SD) RMSE of 122.8 (58.4) mL. On average, females exhibited larger absolute errors and greater variability in the full marker set (absolute difference: 68 vs. 94.3 mL; SD: 19.5 vs. 48.1). However, the difference between males and females was smaller in the reduced marker set (absolute difference: 114.8 mL vs. 95.4 mL).

The linear mixed-effects model showed that OEP and spirometer tidal volumes were strongly related in both marker sets (see Appendix C). For the full marker set, the model yielded a slope of 0.971 (95% CI [0.940, 1.002], t(493) = 61.53, *p* < 0.001; Table 2) and an intercept of −4.62 mL (95% CI [−36.36, 27.13], t(494) = −0.29, *p* = 0.775; Table 2). For the reduced marker set, the slope was significantly below 1 (b = 0.888, 95% CI [0.855, 0.921], t(493) = 52.57, *p* < 0.001; Table 2), while the intercept did differ significantly from zero (b = −76.43 mL, 95% CI [−115.58, −37.27], t(494) = −3.84, *p* < 0.001; Table 2). The marginal R^2^ (fixed effects only) was 0.941 for the full marker set and 0.918 for the reduced marker set; conditional R^2^ (fixed and random effects) was 0.975 and 0.967, respectively.

**Figure 6:**
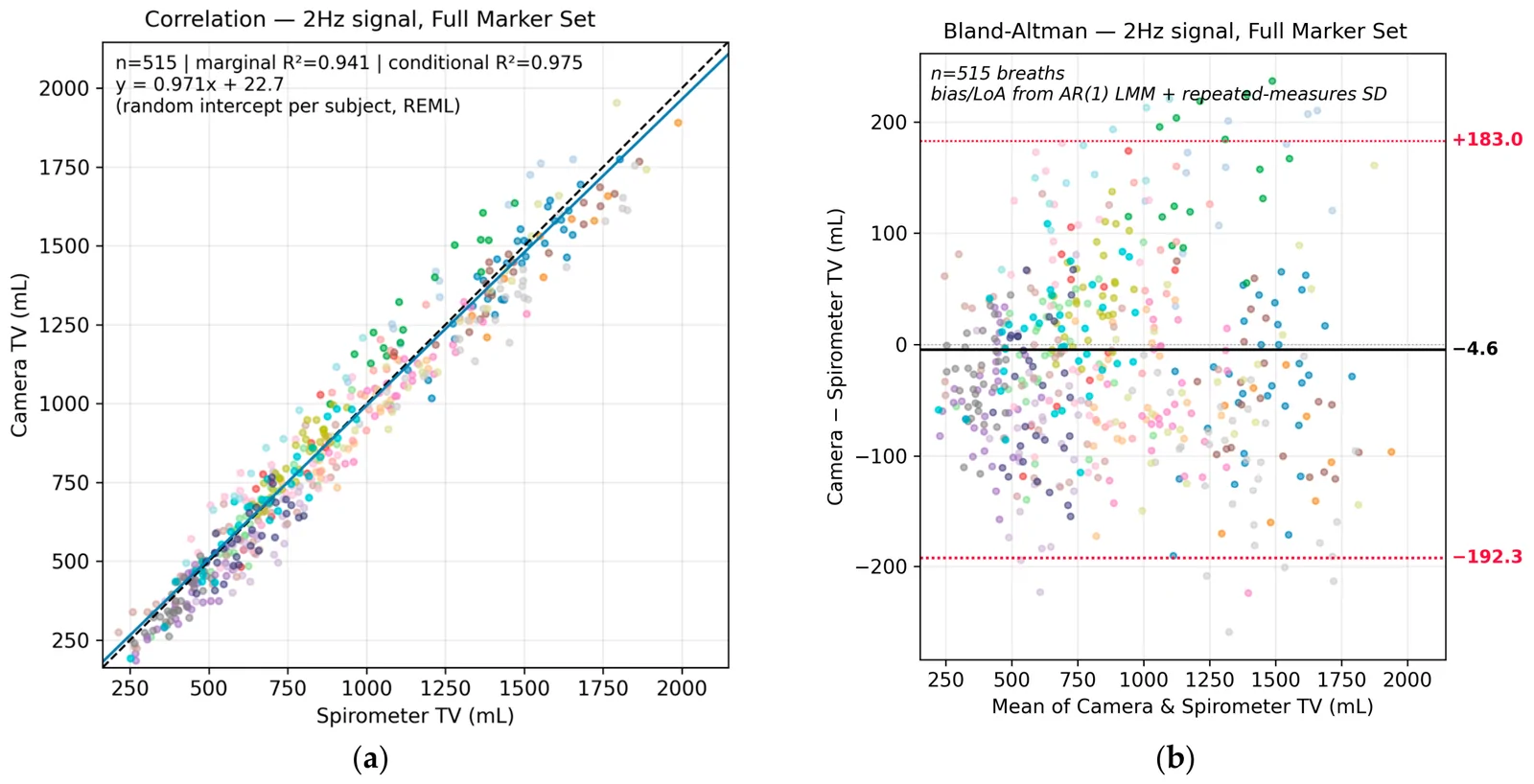
Full marker set. (**a**) Strong, significant correlation between the camera tidal volume (TV) and spirometer TV. Dotted line shows y = x, and the regression line is shown in blue. (**b**) Bland–Altman plot of the camera TV and spirometer TV. Black line indicates the bias, and dotted red lines represent the 95% limits of agreement.

**Figure 7:**
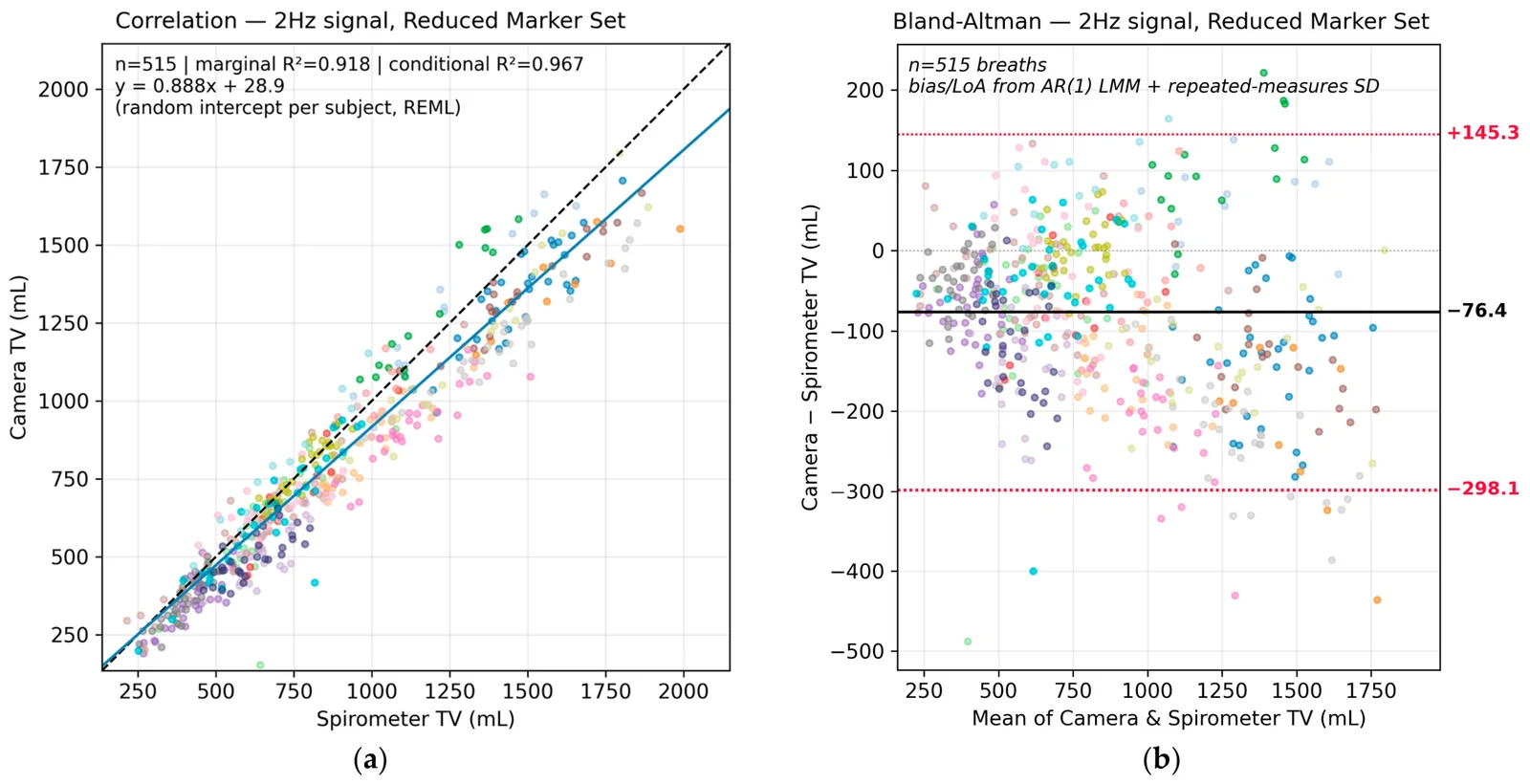
Reduced marker set. (**a**) Strong, significant correlation between the camera method TV and spirometer TV. Dotted line shows the line y = x, and the blue line shows the regression line. (**b**) Bland–Altman plot of the camera method TV and spirometer TV (*n* = 515). Black line indicates the bias, and dotted red lines represent the 95% limits of agreement.

Variances for the full marker set were 5175 mL^2^ and 3990 mL^2^ for between-subject and within-subject, respectively. These variances resulted in a total standard deviation of 95.7 mL. Consequently, the Bland–Altman repeated measures analysis for the full marker set had limits of agreement at −192.3 and 183 mL (Figure 6b). In contrast, the reduced marker set had higher between-subject (7992 mL^2^) and within-subject variances (4801 mL^2^) resulting in a larger total standard deviation (113.1 mL). The reduced marker set had limits of agreement at −298.1 and 145.3 mL (Figure 7b). The Bland–Altman plot for the reduced marker set (Figure 7b) showed a trend for larger errors as volume increased; see Appendix D for further details.

**Figure 8:**
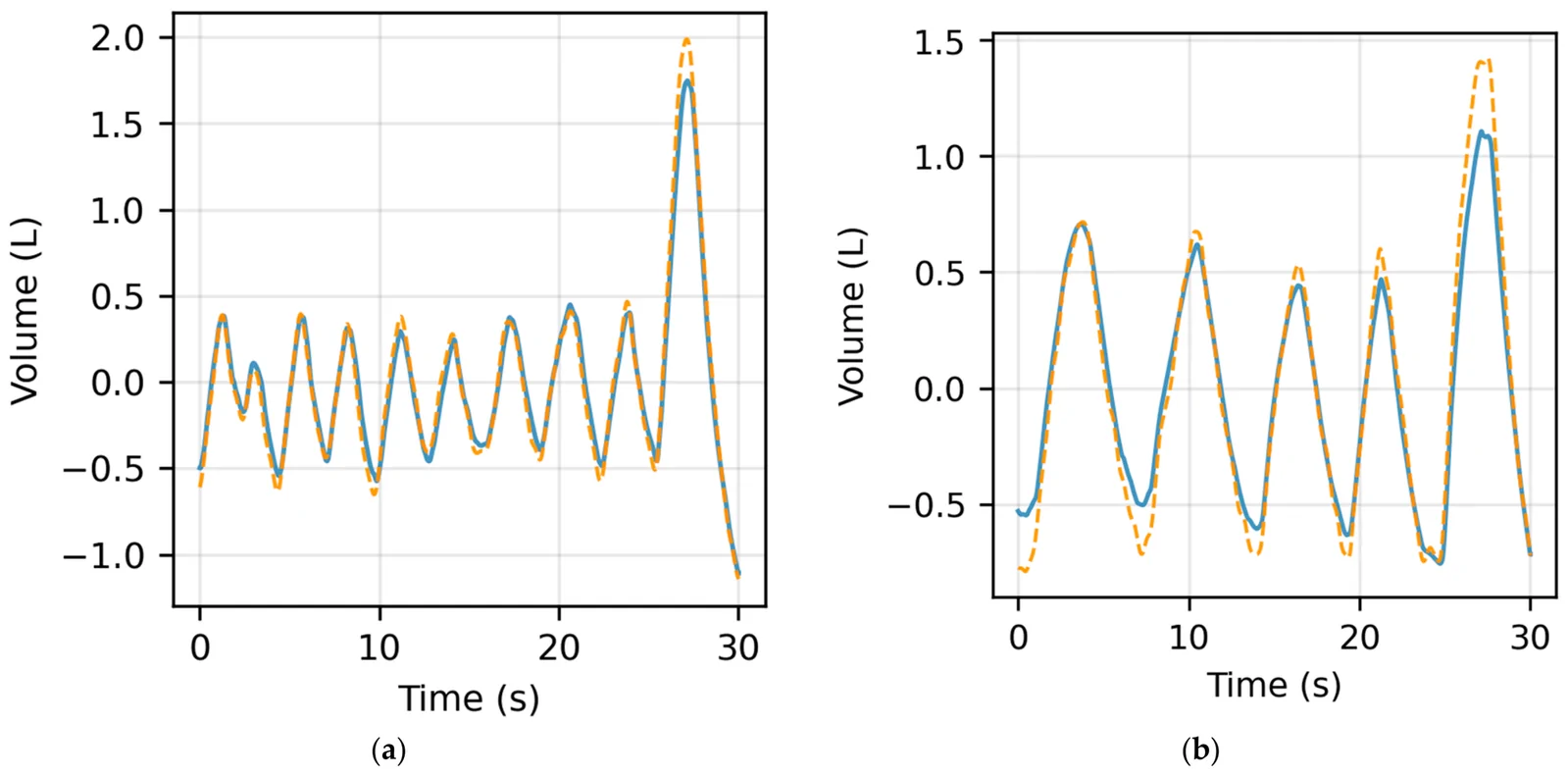

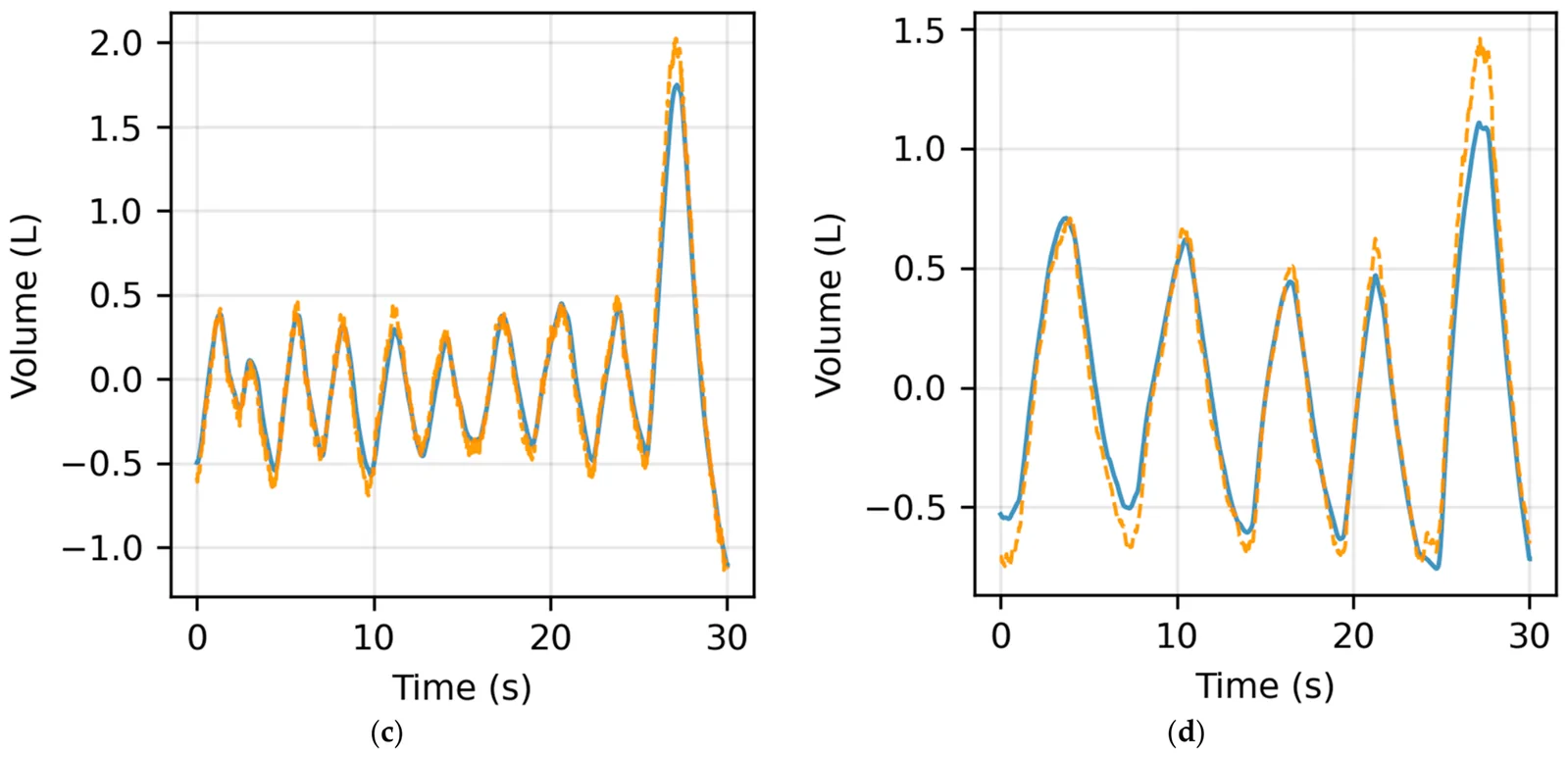
Volume traces obtained from the spirometer (blue) and camera (orange) for two participants: one with good agreement, Spiro21 (**a**,**c**), and one with poor agreement, Spiro3 (**b**,**d**). Data from the full marker set are shown in the upper plots (**a**,**b**), with data from reduced marker set shown in lower plots (**c**,**d**).

Figure 8 shows a comparison of volume–time plots from the camera and spirometer for two participants. The left plots illustrate a participant in which there is a close match between the camera and spirometer, with absolute differences of 39.6 mL (6.6% discrepancy). In contrast, data on the right show lower agreement, with absolute differences of 146.6 mL (11.6%). However, despite the lower agreement in peak-to-peak volume measurement, the lower plots still demonstrate a very close match between the two volume traces.

## 4. Discussion

The aim of this paper was to evaluate the accuracy of tidal volume estimations using a dual RGB-D camera system with two different marker sets. The findings demonstrate that the full 30-marker configuration can accurately estimate volume, showing excellent agreement with spirometer measurements, a low mean bias (−4.6 mL) and narrow limits of agreement (95% LOA: −192.3 to 183.0 mL). However, the 17-marker configuration resulted in a reduction in accuracy (bias: −76 mL, 95% LOA −298 to 145 mL). Overall, these data show that it is possible to accurately estimate tidal volumes using two RGB-D cameras with a configuration of markers which could be used to segment the torso and therefore characterise breathing pattern. However, accuracy is compromised when the number of markers is reduced. Nevertheless, while the 17-marker set may not be appropriate for precise volume estimation, it may be suitable for biofeedback applications designed to convey the salient characteristics of breathing.

Previous studies have compared tidal volume estimates derived from motion capture camera systems with spirometry. When using commercial motion capture systems consisting of 8 to 10 cameras, previously reported biases range from 12 to 70 mL with dispersions between approximately ±120 and ±270 mL, representing ± 1.96 × SD of the differences [4,5]. These findings are comparable to those obtained in the present study for the full marker set, which demonstrated a small bias and similar dispersion (−4.6 ± 187.6 mL). These data suggest that by using a system of two inexpensive RGB-D cameras, it may be possible to measure respiratory volumes during tidal breathing to a similar level of precision as obtained with a laboratory-grade motion capture system.

It is interesting to compare the findings of this study with those of Soleimani et al., [23] who also used two facing depth cameras to estimate respiratory volumes. Soleimani et al. demonstrated that two cameras delivered improved estimates of tidal breathing in standing when compared with one camera. However, their two-camera configuration required individual calibration using spirometry. With this calibration step, they reported a mean (SD) RMSE of 148 (150) mL, which is larger than our RMSE of 90.2 (34.4) mL. Encouragingly, our limits of agreement (−192.3, 183 mL) were also tighter than those reported by Soleimani et al. (−400, 400 mL). This improved accuracy is encouraging, but differences in study population, hardware specifications and processing pipeline limit our abilities to directly compare these studies. Future research could directly compare a marker-based approach to a full point cloud using a common dataset to establish the relative contribution of individual points to volume estimation. Such work may help optimise marker placement or further simplify the marker set. As explained earlier, using a set of markers will allow for segmentation of the torso, thereby enabling characterisation of breathing patterns [30]. These advantages motivate further research that should explore the potential to use 3D camera technology in clinical and exercise testing settings.

Depth camera technology is advancing rapidly and therefore measurement precision is likely to improve. For this study, we used the Intel L515 RGB-D camera, which measures depth using time-of-flight principles. RealSense reports an error in depth measurement of < 5mm with variations of 2.5 mm at 1 m [25]. Despite this relatively large uncertainty, we were still able to accurately estimate tidal volumes, and this is most likely because of the averaging process inherent in the volume calculation. Our dual-camera setup could not capture lateral torso movements directly and therefore relied on a regression model to estimate side-marker motion. However, this approach was developed and validated using the same mannequin, which may have limited its generalisability to females. This is not the only factor that may have contributed to the higher errors observed in the female participants: male participants were unclothed from the waist up, whereas female participants wore a sports bra (Section 2.4), and sex was therefore confounded with clothing in this study. Within OEP, studies using marker-based methods have reported comparable limits of agreement between male and female participants [4,5], although sample sizes are also small. However, when using a whole point cloud rather than markers, it has been found that adding a T-shirt changed the accuracy between males and females [19]. The present study cannot determine mechanism of the increased variability in females. Nevertheless, the strong agreement with spirometry suggests that the overall methodology is promising. Future work should investigate whether higher-precision depth cameras and more sophisticated modelling approaches can further improve accuracy. Current guidelines suggest errors in spirometry should be less than 3% [1]. If this level of accuracy could be achieved with two facing depth cameras, then this may open the door to non-contact respiratory assessment in clinical settings.

There were some further limitations to this study that future research should seek to address. Firstly, this study focused on a young healthy population, with relatively low body mass index. This limits the generalisability of the findings, and therefore further research is needed to investigate the potential for volume estimation across the full spectrum of human body shapes. A second limitation is that participants were required to be unclothed from the waist upwards. This may not be acceptable in some settings, and future research is needed to investigate whether respiratory volumes could be estimated through tight-fitting clothing. Finally, we focused on tidal breathing during relaxed standing. It is therefore unclear whether volume estimation would be possible during dynamic activities, such as walking, running or cycling. If this technology is to be used for clinical respiratory monitoring, such as cardiopulmonary exercise testing, then further research is required to explore performance under dynamic conditions.

## 5. Conclusions

This study demonstrates that it is possible to accurately estimate tidal volumes during relaxed breathing using a dual-depth-camera system. This work highlights the potential for camera-based systems to be used for non-contact respiratory assessment, and further work should explore clinical applications of this technology.

## Figures and Tables

**Table 1:** The breakdown of number of breaths analysed for each participant (*n*) and the participant’s mean absolute difference, absolute discrepancy, normalised Euclidean d and RMSE for the full marker set and the reduced marker set. Male and female data have been presented separately along with a mean (SD) of the full 21 participants.

			Full Marker Set				Reduced Marker Set			
ID	Sex	*n*	Abs Difference (mL)	Abs Discrepancy (%)	Norm. Euclidean d	RMSE (mL)	Abs Difference (mL)	Abs Discrepancy (%)	Norm. Euclidean d	RMSE (mL)
Spiro2	M	32	58.0	4.1	0.003	74.0	133.3	9.1	0.013	154.2
Spiro5	M	21	54.1	5.6	0.005	65.9	150.0	15.2	0.031	159.1
Spiro7	M	31	42.6	6.6	0.008	52.9	67.2	10.2	0.090	111.0
Spiro9	M	13	80.2	8.1	0.008	90.4	76.7	8.0	0.009	86.8
Spiro10	M	33	56.4	13.2	0.031	67.5	74.1	17.1	0.048	84.1
Spiro11	M	30	94.3	14.5	0.036	109.2	128.1	19.7	0.067	144.4
Spiro12	M	20	65.9	4.4	0.003	74.9	132.7	8.7	0.010	146.1
Spiro13	M	39	57.5	10.5	0.017	71.5	64.8	11.4	0.022	78.7
Spiro14	M	27	64.5	5.6	0.005	82.2	216.8	18.9	0.049	227.3
Spiro15	M	26	84.6	13.1	0.021	96.6	70.9	10.4	0.016	84.0
Spiro17	M	32	110.5	7.6	0.008	127.4	225.0	15.5	0.031	237.0
Spiro19	M	30	47.0	6.0	0.005	58.7	26.0	3.3	0.002	34.0
Spiro20	M	22	68.8	5.4	0.004	82.1	127.1	10.2	0.015	143.0
**Mean (M)**		**27.4**	**68.0**	**8.1**	**0.012**	**81.0**	**114.8**	**12.1**	**0.031**	**130.0**
**SD (M)**		**6.9**	**19.5**	**3.6**	**0.011**	**20.7**	**59.5**	**4.8**	**0.026**	**58.7**
Spiro3	F	12	146.6	11.6	0.013	155.4	67.1	5.4	0.003	76.7
Spiro4	F	9	94.3	5.9	0.005	107.4	226.8	13.9	0.026	247.4
Spiro6	F	17	143.1	12.4	0.016	155.9	94.3	7.9	0.007	111.5
Spiro8	F	11	74.9	10.4	0.014	86.4	73.9	10.3	0.021	87.1
Spiro16	F	34	38.6	9.7	0.017	47.9	43.3	11.1	0.024	52.8
Spiro21	F	35	39.6	6.6	0.008	49.6	57.5	9.0	0.024	87.2
Spiro22	F	11	154.4	21.9	0.040	160.5	81.9	11.6	0.014	93.6
Spiro24	F	30	63.0	9.9	0.017	77.4	118.8	18.7	0.055	133.1
**Mean (F)**		**19.9**	**94.3**	**11.0**	**0.016**	**105.1**	**95.4**	**11.0**	**0.022**	**111.2**
**SD (F)**		**11.2**	**48.1**	**4.9**	**0.011**	**47.3**	**57.8**	**4.0**	**0.016**	**59.9**
**Mean** **SD**			**78.0**	**9.2**	**0.014**	**90.2**	**107.4**	**11.7**	**0.027**	**122.8**
			**34.8**	**4.3**	**0.011**	**34.4**	**58.2**	**4.4**	**0.023**	**58.4**

**Table 2:** Fixed-effect estimates from the AR(1)-corrected difference model (diff ~ 1, random intercept + AR(1) residual correlation). B = fixed-effect estimate; SE = standard error; t = t-statistic; CI = confidence interval.

Marker Set	Term	b	SE	t	*p*	95% CI
Full	Intercept (bias, mL)	−4.62	16.16	−0.29	0.775	[−36.36, 27.13]
Full	Slope (spiro TV)	0.971	0.0158	61.53	<0.001	[0.940, 1.002]
Reduced	Intercept (bias, mL)	−76.43	19.93	−3.84	<0.001	[−115.58, −37.27]
Reduced	Slope (spiro TV)	0.888	0.0169	52.57	<0.001	[0.855, 0.921]

**Figure A1:**
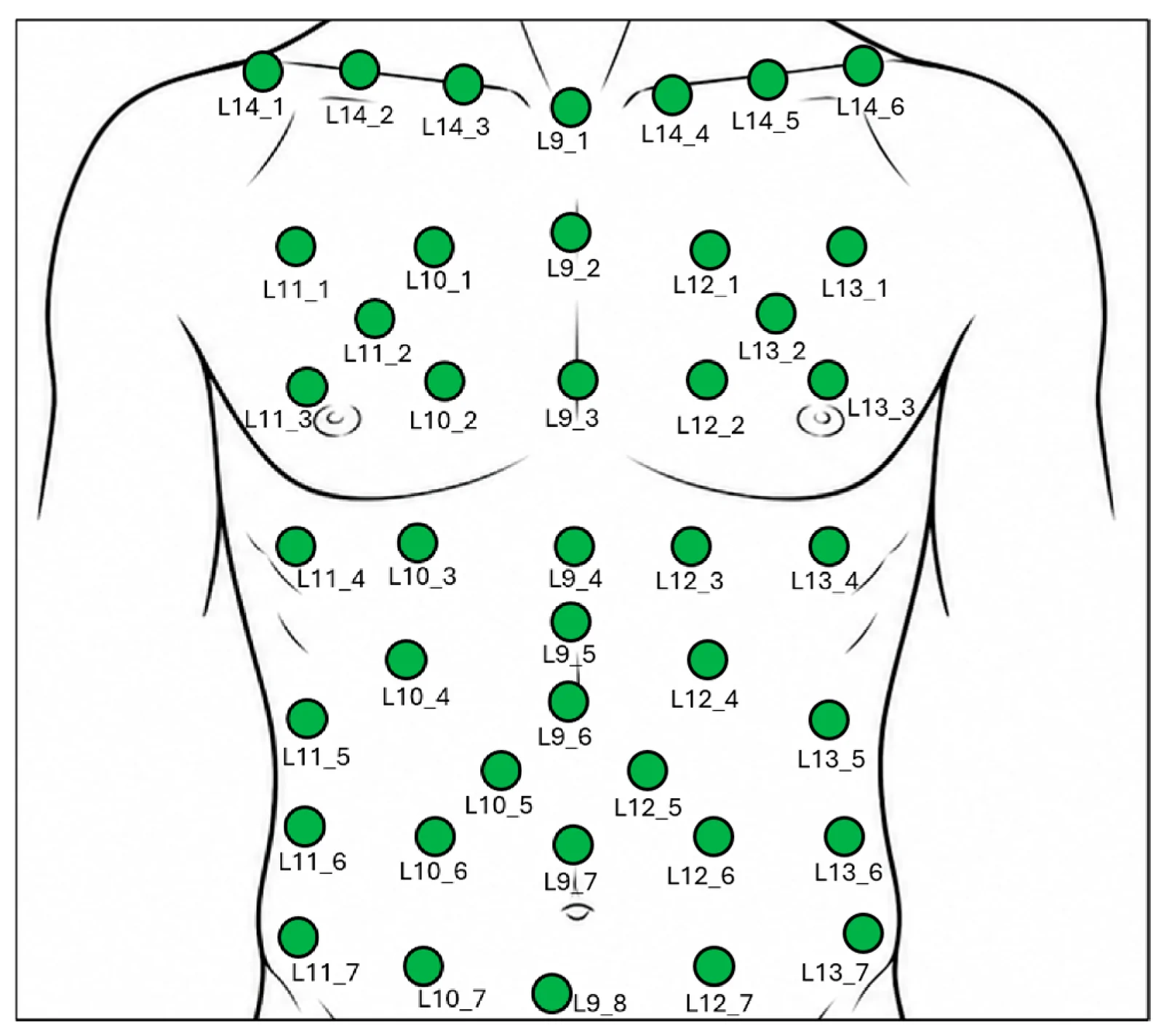
Front markers (physical and virutal).

**Figure A2:**
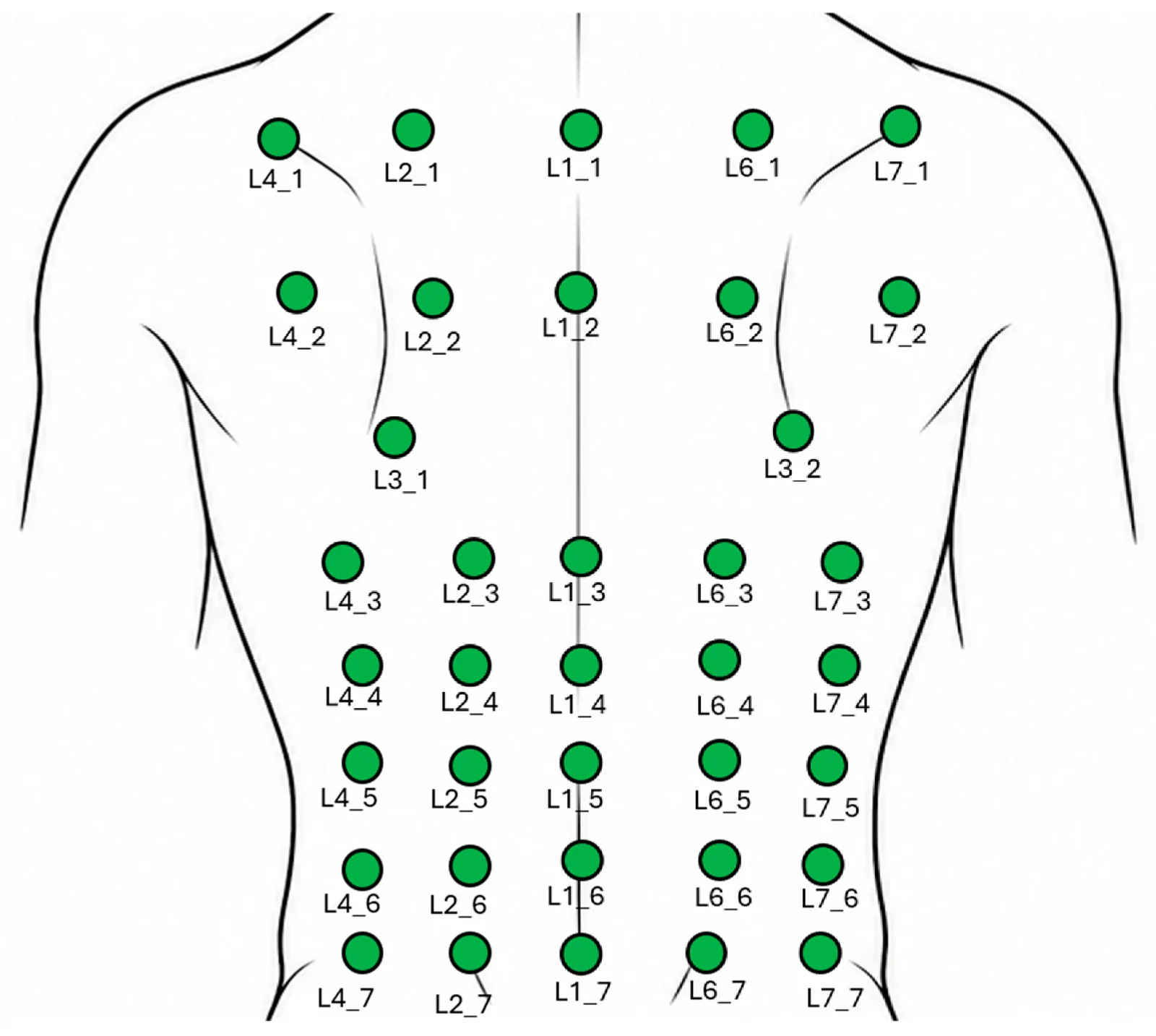
Back markers (physical and virutal).

**Figure A3:**
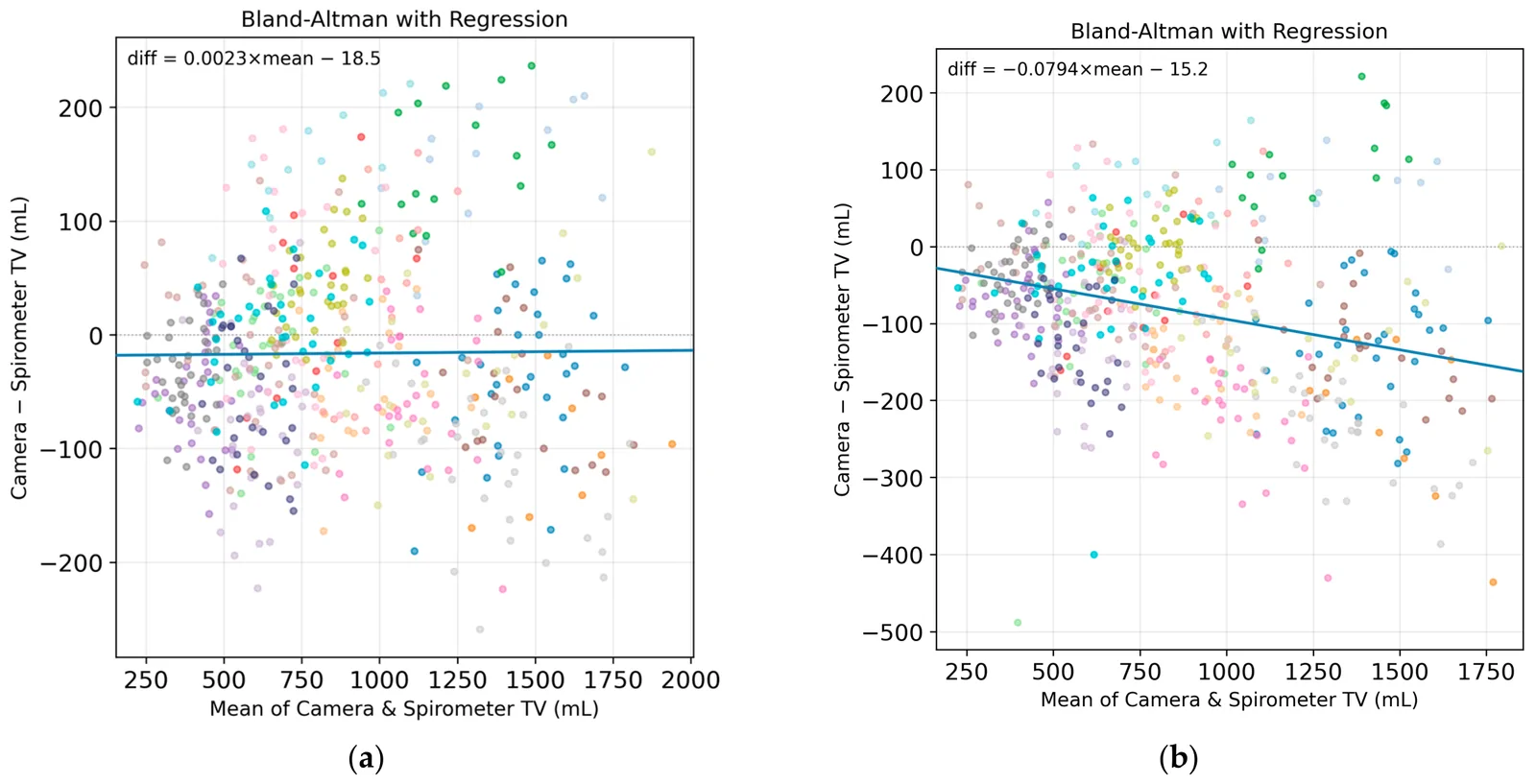
The left plot (**a**) shows the regression line fitted through the full marker set. The right plot (**b**) shows the regression line fitted through the reduced marker set.

## Data Availability

The data presented in this study are available at https://doi.org/10.17866/rd.salford.32587968.

## Abbreviations

The following abbreviations are used in this manuscript:

RMSE	Root Mean Square Error
TV	Tidal Volume
OEP	Optoelectronic Plethysmography
SD	Standard Deviation

## Appendix A. Marker Interpolation

**Table A1:** Interpolation equations for the front markers.

Label	Marker Type		Equation/Placement
	Full Set	Reduced Set	
L9_1	Physical	Physical	Intrajugular notch
L14_1	Physical	Physical	End of right clavicle
L14_6	Physical	Physical	End of left clavicle
L11_7	Physical	Physical	Right ASIS
L9_8	Physical	Physical	Midline of the body on the lowest point of the abdomen
L13_7	Physical	Physical	Left ASIS
L9_4	Physical	Physical	Xiphoid Process
L11_4	Physical	Physical	Level with the Xiphoid Process and in line with right ASIS
L11_5	Physical	Physical	Costal Margin in line with the right ASIS
L13_4	Physical	Physical	Level with the Xiphoid Process and in line with left ASIS
L13_5	Physical	Physical	Costal Margin in line with the left ASIS
L10_4	Physical	Physical	Costal margin at the right mid-clavicular line
L12_4	Physical	Physical	Costal margin at the left mid-clavicular line
L9_2	Physical	Virtual	$\frac{1}{3}(L9\_4)+\frac{2}{3}(\;L9\_1)\;$
L9_3	Physical	Virtual	$\frac{2}{3}(L9\_4)+\frac{1}{3}(\;L9\_1)$
L11_1	Physical	Virtual	$\frac{1}{3}(L11\_4)+\frac{2}{3}(\;L14\_1)$
L11_3	Physical	Virtual	$\frac{2}{3}(L11\_4)+\frac{1}{3}(\;L14\_1)$
L13_1	Physical	Virtual	$\frac{1}{3}(L13\_4)+\frac{2}{3}(\;L14\_6)$
L13_3	Physical	Virtual	$\frac{2}{3}(L13\_4)+\frac{1}{3}(\;L14\_6)$
L14_2	Virtual	Virtual	$\frac{1}{3}(L9\_1)+\frac{2}{3}(\;L14\_1)$
L14_3	Virtual	Virtual	$\frac{2}{3}(L9\_1)+\frac{1}{3}(\;L14\_1)$
L14_4	Virtual	Virtual	$\frac{2}{3}(L9\_1)+\frac{1}{3}(\;L14\_6)$
L14_5	Virtual	Virtual	$\frac{1}{3}(L9\_1)+\frac{2}{3}(\;L14\_6)$
L10_1	Virtual	Virtual	$\frac{1}{2}(L9\_2)+\frac{1}{2}(\;L11\_1)$
L12_1	Virtual	Virtual	$\frac{1}{2}(L9\_2)+\frac{1}{2}(\;L13\_1)$
L10_2	Virtual	Virtual	$\frac{1}{2}(L9\_3)+\frac{1}{2}(\;L11\_3)$
L12_2	Virtual	Virtual	$\frac{1}{2}(L9\_3)+\frac{1}{2}(\;L13\_3)$
L10_3	Virtual	Virtual	$\frac{1}{2}(L9\_4)+\frac{1}{2}(\;L11\_4)$
L12_3	Virtual	Virtual	$\frac{1}{2}(L9\_4)+\frac{1}{2}(\;L13\_4)$
L11_2	Virtual	Virtual	$\frac{1}{2}(L10\_1)+\frac{1}{2}(\;L11\_3)$
L13_2	Virtual	Virtual	$\frac{1}{2}(L12\_1)+\frac{1}{2}(\;L13\_3)$
L10_7	Virtual	Virtual	$\frac{1}{2}(L9\_8)+\frac{1}{2}(\;L11\_7)$
L12_7	Virtual	Virtual	$\frac{1}{2}(L9\_8)+\frac{1}{2}(\;L13\_7)$
L11_6	Virtual	Virtual	$\frac{1}{2}(L11\_7)+\frac{1}{2}(\;L11\_5)$
L13_6	Virtual	Virtual	$\frac{1}{2}(L13\_7)+\frac{1}{2}(\;L13\_5)$
L10_6	Virtual	Virtual	$\frac{3}{4}(L11\_6)+\frac{1}{4}(\;L13\_6)$
L9_7	Virtual	Virtual	$\frac{1}{2}(L11\_6)+\frac{1}{2}(\;L13\_6)$
L12_6	Virtual	Virtual	$\frac{1}{4}(L11\_6)+\frac{3}{4}(\;L13\_6)$
L9_5	Virtual	Virtual	$\frac{2}{3}(L9\_4)+\frac{1}{3}(\;L9\_7)$
L9_6	Virtual	Virtual	$\frac{1}{3}(L9\_4)+\frac{2}{3}(\;L9\_7)$
L10_5	Virtual	Virtual	$\frac{1}{2}(L9\_6)+\frac{1}{2}(\;L10\_6)$
L12_5	Virtual	Virtual	$\frac{1}{2}(L9\_6)+\frac{1}{2}(\;L12\_6)$

**Table A2:** Interpolation Equation Table for the Back Markers.

Label	Marker Type		Placement/Equation
	Full Set	Reduced Set	
L4_1	Physical	Physical	Directly posterior to the end of the left clavicle
L7_1	Physical	Physical	Directly posterior to the end of the right clavicle
L4_7	Physical	Physical	At the level of the PSIS on the left posterior auxillary line
L7_7	Physical	Physical	At the level of the PSIS on the line of the
L1_1	Physical	Physical	$\frac{1}{2}(L4\_1)+\frac{1}{2}(\;L7\_1)$
L1_7	Physical	Virtual	$\frac{1}{2}(L4\_7)+\frac{1}{2}(\;L7\_7)$
L2_7	Physical	Virtual	Left PSIS/$\frac{1}{2}(L4\_7)+\frac{1}{2}(\;L1\_7)$
L6_7	Physical	Virtual	Right PSIS/$\frac{1}{2}(L1\_7)+\frac{1}{2}(\;L7\_7)$
L4_3	Physical	Virtual	$\frac{1}{2}(L4\_7)+\frac{1}{2}(\;L4\_1)$
L1_3	Physical	Virtual	$\frac{1}{2}(L1\_7)+\frac{1}{2}(\;L1\_1)$
L7_3	Physical	Virtual	$\frac{1}{2}(L7\_7)+\frac{1}{2}(\;L7\_1)$
L2_1	Virtual	Virtual	$\frac{1}{2}(L1\_1)+\frac{1}{2}(\;L4\_1)$
L6_1	Virtual	Virtual	$\frac{1}{2}(L1\_1)+\frac{1}{2}(\;L7\_1)$
L2_3	Virtual	Virtual	$\frac{1}{2}(L1\_3)+\frac{1}{2}(\;L4\_3)$
L6_3	Virtual	Virtual	$\frac{1}{2}(L1\_3)+\frac{1}{2}(\;L7\_3)$
L4_2	Virtual	Virtual	$\frac{1}{3}(L4\_3)+\frac{2}{3}(\;L4\_1)$
L2_2	Virtual	Virtual	$\frac{1}{3}(L2\_3)+\frac{2}{3}(\;L2\_1)$
L1_2	Virtual	Virtual	$\frac{1}{3}(L1\_3)+\frac{2}{3}(\;L1\_1)$
L6_2	Virtual	Virtual	$\frac{1}{3}(L6\_3)+\frac{2}{3}(\;L6\_1)$
L7_2	Virtual	Virtual	$\frac{1}{3}(L7\_3)+\frac{2}{3}(\;L7\_1)$
L3_1	Virtual	Virtual	$\frac{1}{2}(L2\_2)+\frac{1}{2}(\;L4\_3)$
L3_2	Virtual	Virtual	$\frac{1}{2}(L6\_2)+\frac{1}{2}(\;L7\_3)$
L4_4	Virtual	Virtual	$\frac{3}{4}(L4\_3)+\frac{1}{4}(\;L4\_7)$
L2_4	Virtual	Virtual	$\frac{3}{4}(L2\_3)+\frac{1}{4}(\;L2\_7)$
L1_4	Virtual	Virtual	$\frac{3}{4}(L1\_3)+\frac{1}{4}(\;L1\_7)$
L6_4	Virtual	Virtual	$\frac{3}{4}(L6\_3)+\frac{1}{4}(\;L6\_7)$
L7_4	Virtual	Virtual	$\frac{3}{4}(L7\_3)+\frac{1}{4}(\;L7\_7)$
L4_5	Virtual	Virtual	$\frac{1}{2}(L4\_3)+\frac{1}{2}(\;L4\_7)$
L2_5	Virtual	Virtual	$\frac{1}{2}(L2\_3)+\frac{1}{2}(\;L2\_7)$
L1_5	Virtual	Virtual	$\frac{1}{2}(L1\_3)+\frac{1}{2}(\;L1\_7)$
L6_5	Virtual	Virtual	$\frac{1}{2}(L6\_3)+\frac{1}{2}(\;L6\_7)$
L7_5	Virtual	Virtual	$\frac{1}{2}(L7\_3)+\frac{1}{2}(\;L7\_7)$
L4_6	Virtual	Virtual	$\frac{1}{4}(L4\_3)+\frac{3}{4}(\;L4\_7)$
L2_6	Virtual	Virtual	$\frac{1}{4}(L2\_3)+\frac{3}{4}(\;L2\_7)$
L1_6	Virtual	Virtual	$\frac{1}{4}(L1\_3)+\frac{3}{4}(\;L1\_7)$
L6_6	Virtual	Virtual	$\frac{1}{4}(L6\_3)+\frac{3}{4}(\;L6\_7)$
L7_6	Virtual	Virtual	$\frac{1}{4}(L7\_3)+\frac{3}{4}(\;L7\_7)$

## Appendix B

Exclusion of the lateral surfaces of the body is common in chest wall volume estimations using integration or volume-averaging techniques. This is because accuracy of depth measurements from 3D cameras decreases at oblique viewing angles, and these surfaces become partially or completely occluded from the camera’s field of view. To quantify the impact of excluding these surfaces, we modelled the torso using planar cuts at the lateral extremities and assessed the resulting volume error.

The markers that are positioned on the midaxillary line were projected onto a plane defined by connecting the most lateral markers on the anterior and posterior aspects of the torso (left: L13 and L4 markers; right: L11 and L7 markers). This translation preserved their vertical position, translating them medially. This approach allowed the volume algorithm to be implemented and used to compare volume estimates between the original marker set and a set in which the markers on the midaxillary line had been projected medially. This comparison was performed on three males and three females selected at random.

The mean Euclidean distance of the translated markers was 38 mm. This translation resulted in a mean underestimation in the absolute chest volume of 1.95 L and a mean difference in breathing volumes of 31.3 mL, which corresponded to a relative error of 3.49%.

**Table A3:** Six participants showing the translation distance of the side markers, the volume bias, difference in breath size, average size of the participants’ tidal volume and the percentage discrepancy compared to the spirometer.

Participant	Sex	BMI	Mean Euclidean Distance (mm)	Total Volume Bias (L)	Mean Breath Difference (L)	Mean Spirometer TV (L)	Discrepancy (%)
Spiro17	M	18.8	37.6	−1.726	0.029	1.345	−2.17%
Spiro19	M	20.6	38.6	−1.786	0.031	0.875	−3.56%
Spiro9	M	28.7	40.5	−2.854	0.035	1.127	−3.07%
Spiro16	F	23.7	35.9	−1.614	0.021	0.489	−4.37%
Spiro3	F	20.4	35.9	−1.716	0.041	1.107	−3.66%
Spiro24	F	26.2	39.6	−2.006	0.031	0.748	−4.13%
Mean		23.1	38.0	−1.951	0.031	0.949	−3.49%

## Appendix C. Linear Mixed-Effects Model

### Appendix C.1. Linear Mixed Model Method

To verify whether a random-intercept model was sufficient to account for the repeated measures, we conducted a Ljung analysis. A random-intercept linear mixed model (diff ~ 1, random intercept for participant, restricted maximum likelihood (REML)) was fitted in Python (statsmodel v0.14) to obtain residuals. We then performed residual analysis with lags to establish whether a model with random intercepts for participants was appropriate. For each participant with ≥10 breaths, residuals were ordered by trial and within-trial breath sequence, and lag-1 to lag-5 autocorrelation and partial autocorrelation functions were computed, together with a Ljung–Box test (lag 5).

Given the temporal residual correlation, we fitted a linear mixed model with a random intercept for participants and an AR(1) correlation structure on the within-participant residuals, nested within trial (diff ~ 1, random = ~1 | participant, correlation = corAR1(~breath | participant/trial)). This model was fitted in R (v4.6.1) using the nlme package (v3.1). The AR(1) model was compared against the random-intercept-only model via a likelihood ratio test. The fixed-effect (bias) estimate was materially unchanged by the AR(1) correction relative to the random-intercept-only model, indicating that the correction primarily affects standard errors and inferential precision rather than the point estimate of agreement.

### Appendix C.2. Linear Mixed Model Results

Across the 20 participants with ≥10 breaths (one participant, with nine breaths, was excluded from this diagnostic), mean lag-1 residual autocorrelation from the random-intercept-only model was 0.174 (median 0.171, range −0.129 to 0.664, SD 0.212) for the full marker set and 0.182 (median 0.184, range −0.109 to 0.583, SD 0.185) for the reduced marker set. Autocorrelation decayed toward zero by lag 3 in both marker sets (mean lag-3 ACF: Full −0.052, Reduced −0.039), consistent with a first-order rather than higher-order autoregressive process. Ljung–Box test at lag 5 was significant (*p* < 0.05) for 3 of 20 participants (15%) in each marker set.

The AR(1) model fitted significantly better than the random-intercept-only model in both marker sets (full: likelihood ratio χ^2^(1) = 26.91, *p* < 0.001, ΔAIC = 31.3, ΔBIC = 27.1; reduced: χ^2^(1) = 27.07, *p* < 0.001, ΔAIC = 25.1, ΔBIC = 20.8). The fitted autoregressive parameter was similar between marker sets (full: φ = 0.297; reduced: φ = 0.258). The fixed-effect (bias) estimate was essentially unchanged by the AR(1) correction (full: −4.51 mL vs. −4.62 mL; reduced: −76.10 mL vs. −76.43 mL, for random-intercept and AR(1), respectively), and standard errors were nearly identical between models (SE ratio AR(1)/random-intercept, full: 0.994; reduced: 1.002). The serial correlation altered the residual covariance structure and model fit without materially changing the estimate of mean bias.

## Appendix D

To investigate whether there was a systematic bias with increasing volume, we fitted an ordinary least squares regression through the Bland–Altman plot (Figure A3) for both the full marker set (Figure A3a) and the reduced marker set (Figure A3b). With the full marker set, the gradient of the regression line was 0.0023 (SE: 0.0097; 95% CI: −0.0168 to 0.0215). The gradient of this line was not significantly different to 0 (*p* = 0.8094). This indicates that there was no proportional bias. In contrast, the reduced marker set regression line had a slope significantly different from 0 (gradient: −0.0794; SE: 0.0117; 95% CI: −0.1924 to −0.0563; *p* < 0.0001), indicating a trend for the camera error to increase with increasing volume.

## References

[1] Graham B.L., Steenbruggen I., Miller M.R., Barjaktarevic I.Z., Cooper B.G., Hall G.L., Hallstrand T.S., Kaminsky D.A., McCarthy K., McCormack M.C. (2019). Standardization of spirometry 2019 update. An official American thoracic society and European respiratory society technical statement. Am. J. Respir. Crit. Care Med..

[2] Wasserman K. (1997). Diagnosing cardiovascular and lung pathophysiology from exercise gas exchange. Chest.

[3] Massaroni C., Carraro E., Vianello A., Miccinilli S., Morrone M., Levai I.K., Schena E., Saccomandi P., Sterzi S., Dickinson J.W. (2017). Optoelectronic Plethysmography in Clinical Practice and Research: A Review. Respiration.

[4] Massaroni C., Senesi G., Schena E., Silvestri S. (2017). Analysis of breathing via optoelectronic systems: Comparison of four methods for computing breathing volumes and thoraco-abdominal motion pattern. Comput. Methods Biomech. Biomed. Eng..

[5] Adhikari N.G., Hunsicker E., Pain M.T.G., Dickinson J.W., Winter S.L. (2025). Agreement Between a Pre-Markered T-Shirt and Manual Marker Placement for Opto-Electronic Plethysmography (OEP) Measures. Sensors.

[6] Smyth C.M.E., Winter S.L., Dickinson J.W. (2021). Novel Real-Time OEP Phase Angle Feedback System for Dysfunctional Breathing Pattern Training—An Acute Intervention Study. Sensors.

[7] Wichum F., Wiede C., Seidl K. (2022). Depth-Based Measurement of Respiratory Volumes: A Review. Sensors.

[8] Addison A.P., Addison P.S., Smit P., Jacquel D., Borg U.R. (2021). Noncontact Respiratory Monitoring Using Depth Sensing Cameras: A Review of Current Literature. Sensors.

[9] Kempfle J., Van Laerhoven K. (2021). Breathing In-Depth: A Parametrization Study on RGB-D Respiration Extraction Methods. Front. Comput. Sci..

[10] Kojima Y., Higaki T., Inoue H., Raytchev B., Gu Y., Nakamura Y. (2026). Contactless Respiratory Waveform Estimation Using a Depth Camera and AI-Based Body Detection. Sensors.

[11] Romano C., Schena E., Silvestri S., Massaroni C. (2021). Non-Contact Respiratory Monitoring Using an RGB Camera for Real-World Applications. Sensors.

[12] Sun C., Li W., Chen C., Wang Z., Chen W. (2019). An Unobtrusive and Non-Contact Method for Respiratory Measurement with Respiratory Region Detecting Algorithm Based on Depth Images. IEEE Access.

[13] Valenzuela A., Sibuet N., Hornero G., Casas O. (2021). Non-Contact Video-Based Assessment of the Respiratory Function Using a RGB-D Camera. Sensors.

[14] Van Hove O., Andrianopoulos V., Dabach A., Debeir O., Van Muylem A., Leduc D., Legrand A., Ercek R., Feipel V., Bonnechère B. (2023). The use of time-of-flight camera to assess respiratory rates and thoracoabdominal depths in patients with chronic respiratory disease. Clin. Respir. J..

[15] Oh K., Shin C.S., Kim J., Yoo S.K. (2019). Level-Set Segmentation-Based Respiratory Volume Estimation Using a Depth Camera. IEEE J. Biomed. Health Inform..

[16] Aoki H., Miyazaki M., Nakamura H., Furukawa R., Sagawa R., Kawasaki H. Non-contact respiration measurement using structured light 3-D sensor. Proceedings of the 2012 Proceedings of SICE Annual Conference (SICE).

[17] L’hEr E., Nazir S., Pateau V., Visvikis D. (2022). Accuracy of noncontact surface imaging for tidal volume and respiratory rate measurements in the ICU. J. Clin. Monit. Comput..

[18] Wijenayake U., Park S.-Y. (2017). Real-Time External Respiratory Motion Measuring Technique Using an RGB-D Camera and Principal Component Analysis. Sensors.

[19] Imano W., Kameyama K., Hollingdal M., Refsgaard J., Larsen K., Topp C., Kronborg S.H., Gade J.D., Dinesen B. (2020). Non-Contact Respiratory Measurement Using a Depth Camera for Elderly People. Sensors.

[20] Addison P.S., Smit P., Jacquel D., Addison A.P., Miller C., Kimm G. (2022). Continuous non-contact respiratory rate and tidal volume monitoring using a Depth Sensing Camera. J. Clin. Monit. Comput..

[21] Bachir W. (2026). Time-of-flight abdominal wall displacement for non-invasive longitudinal monitoring of pulmonary function. Physiol. Meas..

[22] Takamoto H., Nishine H., Sato S., Sun G., Watanabe S., Seokjin K., Asai M., Mineshita M., Matsui T. (2020). Development and Clinical Application of a Novel Non-contact Early Airflow Limitation Screening System Using an Infrared Time-of-Flight Depth Image Sensor. Front. Physiol..

[23] Soleimani V., Mirmehdi M., Damen D., Camplani M., Hannuna S., Sharp C., Dodd J. (2018). Depth-Based Whole Body Photoplethysmography in Remote Pulmonary Function Testing. IEEE Trans. Biomed. Eng..

[24] Sharp C., Soleimani V., Hannuna S., Camplani M., Damen D., Viner J., Mirmehdi M., Dodd J.W. (2017). Toward Respiratory Assessment Using Depth Measurements from a Time-of-Flight Sensor. Front. Physiol..

[25] RealSense Intel RealSense LiDAR Camera L515 Datasheet. https://dev.realsenseai.com/docs/lidar-camera-l515-datasheet/.

[26] RealSense. librealsense. v2.50.0. https://github.com/realsenseai/librealsense.

[27] Massaroni C., Schena E., Bastianini F., Scorza A., Saccomandi P., Lupi G., Botta F., Sciuto S.A., Silvestri S. (2014). Development Of A Bio-Inspired Mechatronic Chest Wall Simulator for Evaluating the Performances of Opto-Electronic Plethysmography. Open Biomed. Eng. J..

[28] Nakagawa S.H. (2013). Schielzeth, A general and simple method for obtaining R2 from generalized linear mixed-effects models. Methods Ecol. Evol..

[29] Bland J.M., Altman D.G. (2007). Agreement Between Methods of Measurement with Multiple Observations Per Individual. J. Biopharm. Stat..

[30] Konno K., Mead J. (1967). Measurement of the separate volume changes of rib cage and abdomen during breathing. J. Appl. Physiol..

